# Genomic characterization of highly pathogenic avian influenza A(H5N1) clade 2.3.4.4b into wild seabirds from coastal Ecuador

**DOI:** 10.3389/fmicb.2026.1953002

**Published:** 2026-09-25

**Authors:** Andrés Carrazco-Montalvo, Lucía Luje, Cristina Rodríguez-Pólit, Antonio Ampuño, Diana Gutiérrez-Pallo, Daniel Alava, Leandro Patiño, Natali Argüello, Gabriela Echeverría-Garcés, David de la Torre, Gabriel da Luz Wallau, Josefina Coloma

**Affiliations:** 1 Instituto de Microbiología, Universidad San Francisco de Quito, Quito, Ecuador; 2 Ministerio de Ambiente y Energía, Quito, Ecuador; 3 Escuela de Ciencias Biológicas e Ingeniería, Universidad Yachay Tech, Quito, Ecuador; 4 Facultad de Ciencias de la Vida, Escuela Superior Politécnica del Litoral, Guayaquil, Ecuador; 5 Facultad de Ciencias de la Salud y Desarrollo Humano, Universidad ECOTEC, Samborondón, Ecuador; 6 Laboratorio Zonal de Influenza y Otros Virus Respiratorios, Instituto Nacional de Investigación en Salud Pública “Dr. Leopoldo Izquieta Pérez”, Quito, Ecuador; 7 Centro de Referencia Nacional de Genómica, Secuenciación y Bioinformática, Instituto Nacional de Investigación en Salud Pública “Dr. Leopoldo Izquieta Pérez”, Quito, Ecuador; 8 Experimental and Applied Biomedicine Research Group, Health Sciences Faculty, Universidad Internacional SEK (UISEK), Quito, Ecuador; 9 Laboratorio de Biología y Genética Molecular—LABIGEN, Quito, Ecuador; 10 Fundação Oswaldo Cruz (FIOCRUZ), Recife, Pernambuco, Brazil; 11 WHO Collaborating Center for Arbovirus and Hemorrhagic Fever Reference and Research, National Reference Center for Tropical Infectious Diseases, Bernhard Nocht Institute for Tropical Medicine, Hamburg, Germany; 12 Universidade Federal de Santa Maria (UFSM), Santa Maria, Rio Grande do Sul, Brazil; 13 Division of Infectious Diseases and Vaccinology, School of Public Health, University of California Berkeley, Berkeley, CA, United States

**Keywords:** clade 2.3.4.4b, Ecuador, genomic epidemiology, H5N1, highly pathogenic avian influenza, one health, wild birds, genomic surveillance

## Abstract

**Introduction:**

Highly pathogenic avian influenza A(H5N1) clade 2.3.4.4b has spread widely across South America, but its genomic diversity and patterns of introduction into Ecuador remain poorly characterized.

**Methods:**

In this study, we report and characterize four genomes of A(H5N1) viruses detected in four individuals of two Ecuadorian seabird species: two magnificent frigatebirds (*Fregata magnificens*) and two blue-footed boobies (*Sula nebouxii*) sampled in 2023.

**Results:**

These genomes represent confirmed detections of avian influenza in wild birds in the country. The viruses were assigned to genotype B3.2. Global phylogenetic analysis showed that the Ecuadorian wild-bird viruses formed groups distinct from viruses detected in domestic birds in Ecuador. Subsequent phylogenetic and phylogeographic analyses within the South American dataset placed our samples in two distinct clusters, each closely related to viruses previously detected in Peru. These findings highlight the genetic diversity of genotype B3.2 viruses detected in Ecuador and their relationship to viruses circulating in the region. FluServer screening identified 29 amino acid substitutions previously associated with virulence, antigenic change, host specificity, polymerase activity, or antiviral resistance.

**Discussion:**

These findings provide genomic evidence of repeated H5N1 introductions into Ecuador as part of the regional spread of the virus along the eastern Pacific coast. They also support sustained surveillance across wildlife, animal-health, and public-health sectors.

## 1 Introduction

Highly pathogenic avian influenza HPAI-A(H5N1) viruses are a major global concern because of their impacts on animal health, biodiversity, and poultry production, as well as their potential risk to human health. These viruses have been recognized for their pandemic potential since the early 2000s (Peiris et al., 2007). The ancestral A/goose/Guangdong/1/1996 virus was first identified in domestic geese in Guangdong Province, China, in 1996 and later diversified into several genetic clades and genotypes (Xu et al., 1999). Among them, clade 2.3.4.4b has reached an unprecedented geographic and host range. Since 2020, viruses from this clade have spread across Europe, Africa, Asia, and the Americas, reaching North America through transatlantic movements of wild birds in late 2021 (Wille and Barr, 2022; Caliendo et al., 2022; Xie et al., 2023; Bellido-Martín et al., 2026). Over the course of the current panzootic, A(H5N1) viruses have caused extensive outbreaks in poultry, unprecedented mortality among wild birds, and an increasing number of infections in mammalian species (Couty et al., 2026; Bellido-Martín et al., 2026). Their circulation in wild-bird populations and movement along interconnected migratory flyways have contributed to the long-distance spread of the virus and its introduction into previously unaffected regions (Couty et al., 2026; Bellido-Martín et al., 2026). HPAI-A(H5N1) activity remains widespread: between 2025 and 2026, more than 2,000 outbreaks were reported by 64 countries and territories, resulting in the death or culling of more than 140 million poultry (World Organisation for Animal Health, 2026).

Human infections with HPAI-A(H5N) remain uncommon despite the virus’s widespread circulation in animals, but they continue to highlight its enzootic and zoonotic potential. As of July 7, 2026, the World Health Organization had recorded 1,000 laboratory-confirmed human infections with A(H5N1), including 479 deaths, since 2003 (World Health Organization, 2026a). Most infections have occurred after direct or indirect exposure to infected animals or contaminated environments; sustained human-to-human transmission has not been documented (World Health Organization, 2026a,b).

The expansion of H5N1 clade 2.3.4.4b into South America marked a new stage of the panzootic. The virus was first detected in the region in Colombia in October 2022, then spread rapidly through several countries (Leguia et al., 2023; Artuso et al., 2025). Its arrival was followed by outbreaks in poultry and extensive mortality among wild birds, particularly along the Pacific coast. In Peru and Chile, Peruvian pelicans, boobies, cormorants, and other seabirds were among the most visibly affected species (Leguia et al., 2023; Cruz et al., 2023; Ulloa et al., 2023). The impact was not limited to birds. Infections and mass mortality were also reported in South American sea lions (*Otaria flavescens*) and other marine mammals, revealing the broad host range of the virus and its capacity to affect coastal ecosystems where birds and mammals gather in large numbers (Leguia et al., 2023; Cruz et al., 2023; Ulloa et al., 2023; Rimondi et al., 2024).

Ecuador confirmed HPAI-A(H5N1) outbreaks in poultry and wild birds during this regional expansion. In January 2023, the country also reported the first human infection with an avian influenza A(H5) virus in Latin America and the Caribbean (World Health Organization, 2023). Ecuador lies along the Pacific Americas Flyway and includes continental coastal habitats and oceanic ecosystems that support large populations of migratory and resident seabirds. These populations may contribute to the introduction and movement of avian influenza viruses between breeding, feeding, and resting areas. However, genomic information of viruses sampled from wild birds in Ecuador remains limited, making it difficult to reconstruct viral introduction routes and determine how viruses detected in the country are related to those circulating elsewhere in the Americas.

In this study, through a One Health framework between the National Institute for Public Health Research—Ecuador (INSPI) and the Ministry of Environment and Energy—Ecuador (MAE), we report the detection and genomic characterization of HPAI-A(H5N1) clade 2.3.4.4b viruses from four individuals of two species of Ecuador’s seabirds: two frigatebirds (*Fregata magnificens*) and two blue-footed boobies (*Sula nebouxii*). Using whole-genome sequencing, phylogenetic and phylogeographic analyses, we investigated the origins and spatiotemporal pathways through which these viruses entered the country and assessed their relationships with viruses circulating in other parts of the Americas. We also examined amino acid substitutions previously associated with relevant biological characteristics of HPAI-A(H5N1) viruses.

## 2 Materials and methods

### 2.1 Sample collection

Tracheal swab samples were collected from four individuals of two seabird species in Ecuador: two frigatebirds (*F. magnificens*), one sick and one found dead, sampled in San Vicente, Manabí Province in January 2023; and two sick blue-footed boobies (*S. nebouxii*) sampled in General Villamil Playas, Guayas Province in May 2023 (Figures 1A–C). Sampling was conducted by the Ministry of Environment and Energy (Ministerio de Ambiente y Energía) (Supplementary material 1).

**Figure 1:**
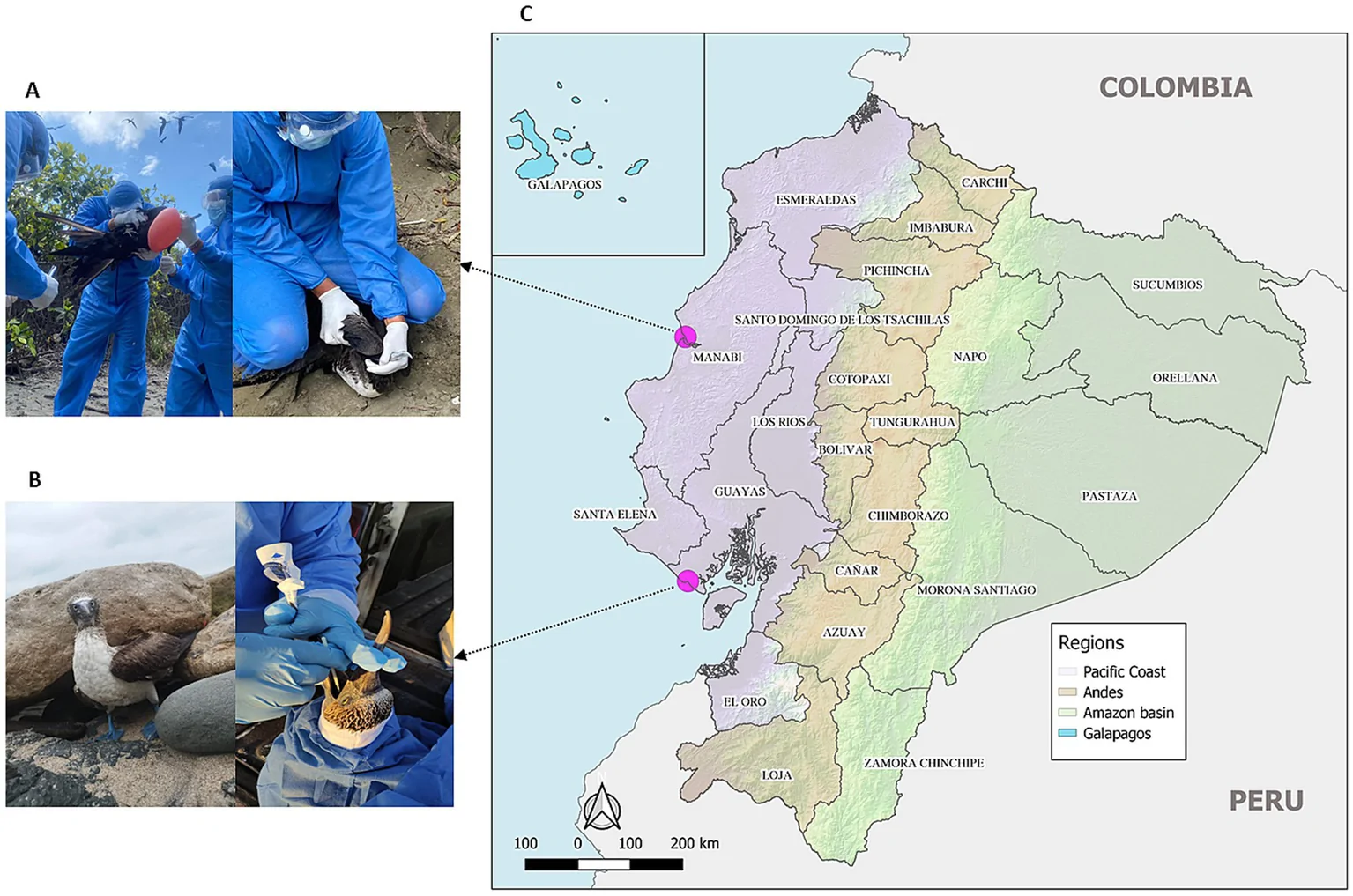
**(A)** Frigatebird (*Fregata magnificens*) sample collection: tracheal swab. **(B)** Blue-footed booby (*Sula nebouxii*) sample collection: tracheal swab. **(C)** Map of Ecuador showing collection points: San Vicente-Manabí and Villamil Playas-Guayas.

### 2.2 Diagnostic confirmation, genotyping, and whole-genome sequencing

Tracheal swab samples were initially tested for influenza A(H5N1) virus by RT-qPCR at LABIGEN, a private laboratory located in Quito. Diagnostic confirmation and genotyping were subsequently performed at the INSPI, Quito headquarters. Samples were confirmed as positive for influenza A(H5) virus by RT-qPCR at the Zonal Laboratory for Influenza and Other Respiratory Viruses. Whole-genome sequencing and bioinformatic analyses were conducted at the National Reference Center for Genomics, Sequencing and Bioinformatics (Centro de Referencia Nacional de Genómica, Secuenciación y Bioinformática, CRN-GENSBIO). Sequencing was performed on an Illumina MiSeq instrument using the Illumina Respiratory Virus Oligo Panel (RVOP) and a MiSeq v2 150-cycle reagent kit (Illumina, Inc., n.d.). The quality of the FASTQ files was evaluated using FastQC (Andrews, 2010), and adapter sequences and low-quality bases were removed using Trimmomatic (Bolger et al., 2014). Filtered reads were mapped using Bowtie2 v2.5.1 (Langmead and Salzberg, 2012) against the eight genomic segments of the influenza A/goose/Guangdong/1/1996 (H5N1) reference virus: PB2 (NC_007357.1), PB1 (NC_007358.1), PA (NC_007359.1), HA (NC_007362.1), NP (NC_007360.1), NA (NC_007361.1), M (NC_007363.1), and NS (NC_007364.1). Consensus sequences were generated from the mapped reads. Four whole-genome sequences, one from each sampled bird, were obtained and deposited in GISAID under accession numbers EPI_ISL_17973443, EPI_ISL_17973458, EPI_ISL_18137626, and EPI_ISL_18137671.

### 2.3 Neuraminidase subtype, clade, and genotype assignment

The neuraminidase subtype was determined by sequence similarity searches using the Basic Local Alignment Search Tool (BLAST) and the Bacterial and Viral Bioinformatics Resource Center (BV-BRC) (Altschul et al., 1990; Olson et al., 2023). Clade assignment was based on phylogenetic analysis of the HA segment. The HA sequences of the Ecuadorian viruses were analyzed together with a reference dataset of 625 avian influenza A(H5) virus sequences retrieved from BV-BRC. This analysis classified the four Ecuadorian viruses as highly pathogenic avian influenza A(H5N1) viruses belonging to clade 2.3.4.4b.

Genotype assignment was conducted using GenoFLU v1.01, which utilizes BLAST to identify each genome segment against a curated reference database and assigns genotypes according to the resulting eight-segment constellation (Youk et al., 2023a). Genotypes were assigned based on similarity calculations.

### 2.4 Exploratory phylogenetic reconstruction using augur

An exploratory phylogenetic analysis was conducted using the Augur bioinformatics pipeline implemented in Nextstrain version 24.3.0 (Huddleston et al., 2021). The dataset included all available highly pathogenic avian influenza A(H5N1) virus sequences deposited in the GISAID database up to 9 September 2026 that corresponded to clade 2.3.4.4b and had been assigned to genotype B3.2.

The analysis was performed using nucleotide sequences from the hemagglutinin (HA) segment. Sequences were filtered to retain only those with at least 90% coverage of the segment. Sequences were excluded if they lacked complete collection-date or sampling-location information, had an embargo status in GISAID, or were flagged as having a “type mismatch,” indicating an inconsistency between the submitted sequence and its associated virus metadata. Following the application of these filters, 2,314 sequences were retained for the exploratory analysis. These sequences contained both sampling-location and collection-date information suitable for phylogenetic and temporal analyses.

The main parameters used throughout the Augur workflow are described below, including those applied during sequence alignment, phylogenetic inference, temporal reconstruction, and visualization. Nucleotide sequences were aligned using MAFFT v7 (Katoh and Standley, 2013). A maximum-likelihood phylogenetic tree was inferred using IQ-TREE v2.2.0 (Minh et al., 2020) under the general time-reversible (GTR) nucleotide substitution model, selected using ModelFinder. Nodal support was assessed using 1,000 bootstrap replicates. The resulting maximum-likelihood topology was converted into a time-scaled phylogeny using TreeTime version 0.11.4 (Sagulenko et al., 2018). Temporal calibration incorporated the sampling dates of the sequences and a fixed evolutionary rate of 0.008 substitutions per site per year. Internal node dates were estimated using marginal inference to reconstruct the temporal emergence and diversification patterns of genotype B3.2 within H5N1 clade 2.3.4.4b. The resulting time-scaled phylogeny, together with the associated geographic metadata and inferred spatiotemporal patterns, was visualized using Auspice. The phylogenetic tree was visualized in RStudio Team (2024).

### 2.5 Molecular clock and ancestral state reconstruction

After completing the exploratory analysis described above, we conducted a more focused analysis by selecting South American sequences that clustered with the Ecuadorian wild-bird sequences reported in this study. The resulting dataset included available sequences from Ecuador, Peru, Chile, Argentina, Uruguay, and other South American countries. We included sequence A/fox/Minnesota/22/016487/001/original/2022 (EPI_ISL_15078249), assigned to genotype B3.2, as a representative sequence for comparison with the South American viruses. Sequences corresponding to the eight influenza A virus genome segments were aligned independently using MAFFT v7 (Katoh and Standley, 2013). The segment-specific alignments were then concatenated to generate a whole-genome alignment. A maximum-likelihood phylogenetic tree was reconstructed using IQ-TREE v2.2.0 (Minh et al., 2020), and the best-fitting nucleotide substitution model was selected with ModelFinder based on the Bayesian information criterion (Kalyaanamoorthy et al., 2017).

The temporal signal of the concatenated dataset was assessed through root-to-tip regression using the maximum-likelihood tree as input in TempEst v1.5.3 (Rambaut et al., 2016). The analysis was conducted using both the selected outgroup root and the best-fitting root identified by TempEst. In both rooting schemes, the regression showed a positive relationship between genetic divergence and sampling time, supporting the use of a molecular-clock model. Bayesian phylogenetic reconstruction was performed using BEAST v1.10.5 (Suchard et al., 2018). To account for variation in evolutionary rates among lineages, an uncorrelated lognormal relaxed molecular clock was used. The analysis incorporated a Bayesian Skygrid coalescent model (Gill et al., 2013) and the GTR + F + G4 nucleotide substitution model, which was selected by ModelFinder. Base frequencies were estimated from the alignment, and among-site rate heterogeneity was modeled using four gamma categories. Sampling dates were assigned to the tips.

The mean evolutionary rate was assigned a lognormal prior with a mean of 0 and a standard deviation of 1.0 on the log scale. The standard deviation of the uncorrelated relaxed-clock rates was assigned an exponential prior with a mean of 1.0. The Skygrid population-size parameters were assigned a 1/x prior. Country was included as discrete traits for ancestral-state and phylogeographic reconstruction following the framework proposed by Lemey et al. (2009). The Markov chain Monte Carlo analysis was run for 100 million generations, with parameters and trees sampled every 10,000 generations. The first 10% of samples were discarded as burn-in. Convergence and mixing were evaluated in Tracer v1.7.2 by inspecting trace plots and confirming effective sample size values greater than 200 for all relevant parameters. A maximum clade credibility tree was summarized using TreeAnnotator v1.1.10.5 and visualized in FigTree v1.4.4 (Rambaut, 2018).

### 2.6 Mutations analysis

SNPs and mutation analyses were performed to identify amino acid changes with potential biological effects related to virulence, immune escape, host specificity, and drug resistance. Each segment was analyzed using FluSurver^1^ and compared to the reference influenza genome previously detailed. Only mutations with previously characterized significant biological effects were included in the mutation list.

## 3 Results

### 3.1 Genome metrics

Genome recovery metrics for the four influenza A(H5N1) genomes are summarized in Table 1. High-depth sequencing data were obtained for all samples, with an average depth of approximately 1,000× in the recovered viral segments. This level of read support allowed for the generation of nearly complete consensus genomes for all viruses, although this varied depending on the segment and sample. Overall segment coverage ranged from 80.0 to 100.0%.

**Table 1:** Recovery metrics for influenza A virus genome segments in sequencing samples.

Segment	Length (bp)	Mean depth (×)	Sample 1 coverage (%)	Sample 2 coverage (%)	Sample 3 coverage (%)	Sample 4 coverage (%)
PB2	2,340	1,000	99.5	100.0	92.5	85.6
PB1	2,341	1,000	98.3	99.3	90.3	83.3
PA	2,233	1,000	99.2	99.1	99.0	98.7
HA	1,751	1,000	99.5	90.5	99.7	80.0
NP	1,565	1,000	100.0	99.6	98.8	99.6
NA	1,458	1,000	98.5	90.3	99.0	98.1
MP	1,027	1,000	99.7	99.4	100.0	100.0
NS	890	1,000	94.8	90.9	—	81.3

Sample identification: Sample 1, *Fregata magnificens* (EPI_ISL_17973443); Sample 2, *Fregata magnificens* (EPI_ISL_17973458); Sample 3, *Sula nebouxii* (EPI_ISL_18137626); and Sample 4, *Sula nebouxii* (EPI_ISL_18137671).

Mean sequencing depth was 1,000 × for all segments. An em dash (—) indicates that coverage was not available or could not be calculated.

The virus detected in the two *F. magnificens* specimens showed consistently high recovery across all eight genomic segments, whereas greater variation in coverage was observed in the viruses obtained from the two *S. nebouxii* specimens. The main limitation was the failure to recover the NS segment from sample 3. Additionally, sample 4 showed lower coverage for some segments, particularly HA. Nevertheless, the recovered sequence data met the predefined quality criteria and provided sufficient genome completeness to support phylogenetic, genotypic, and mutation analyses. The resulting influenza A(H5N1) genomes were deposited in GISAID under accession numbers EPI_ISL_17973443 for sample 1 (*F. magnificens*), EPI_ISL_17973458 for sample 2 (*F. magnificens*), EPI_ISL_18137626 for sample 3 (*S. nebouxii*), and EPI_ISL_18137671 for sample 4 (*S. nebouxii*).

### 3.2 Clade and genotype assignment

Phylogenetic analysis of the hemagglutinin (HA) gene placed all four influenza A(H5N1) viruses detected in Ecuador’s seabirds within clade 2.3.4.4b of the highly pathogenic avian influenza H5 lineage (Figure 2). The sequences detected in Ecuador grouped within the 2.3.4.4b cluster and were phylogenetically distinct from the other clades represented in the analysis, including 2.3.4.4a, 2.3.4.4c, 2.3.4.4d, 2.3.4.4e, 2.3.4.4f, 2.3.4.4g, and 2.3.4.4h. This clustering supported a consistent HA clade assignment for viruses detected in both *F. magnificens* and *S. nebouxii*.

**Figure 2:**
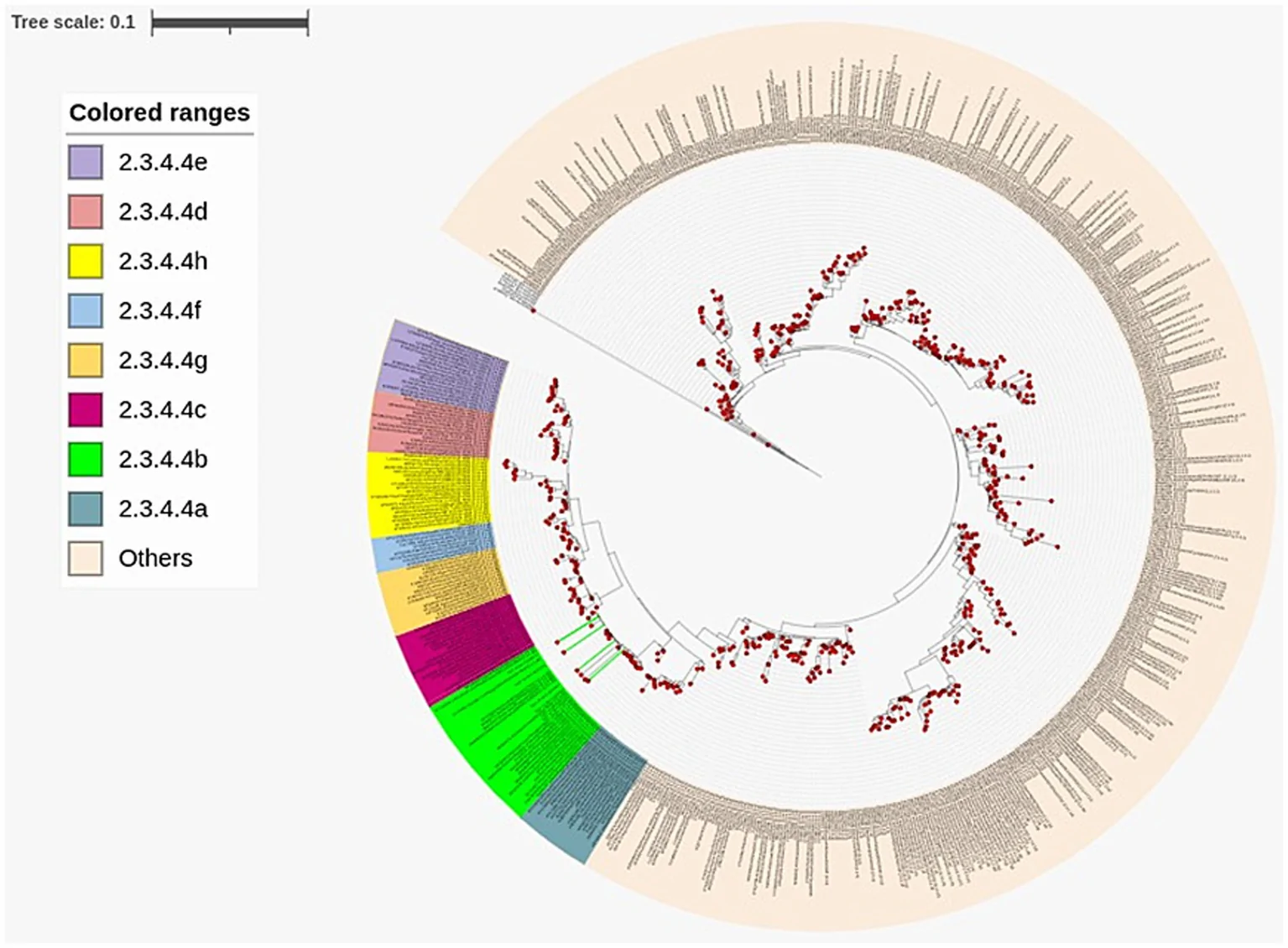
Phylogenetic tree of the hemagglutinin (HA) segment of avian influenza A(H5) viruses. Clade 2.3.4.4b assignment is highlighted in light green. Samples from Ecuador are indicated with light green branch lines.

Whole-genome genotype assignment was performed using GenoFLU based on the segment-group classification of the eight influenza A virus gene segments. Genome constellations were reported in the order PB2|PB1|PA|HA|NP|NA|M|NS. Samples 1, 2, and 4 showed the same complete segment-group constellation, am2.1|am1.2|ea1|ea1|am1.4.1|ea1|ea1|am1.1, corresponding to genotype B3.2 (Table 2). For Sample 3, the NS segment was not recovered; therefore, the NS segment group could not be assigned. Its genotype classification was reported only based on the seven available segments. No differences in genome constellation were observed between viruses obtained from *F. magnificens* collected in January 2023 and *S. nebouxii* collected in May 2023. Thus, although the samples were separated by host species and collection date, the available genomic evidence did not indicate the circulation of distinct reassorting genotypes among the analyzed samples.

**Table 2:** Influenza A virus segment-group assignments, genome constellations, and genotypes for the four avian samples.

Sample	Segment group								Genome constellation	Genotype assignment
	PB2	PB1	PA	HA	NP	NA	M	NS		
1	am2.1	am1.2	ea1	ea1	am1.4.1	ea1	ea1	am1.1	am2.1|am1.2|ea1|ea1|am1.4.1|ea1|ea1|am1.1	B3.2
2	am2.1	am1.2	ea1	ea1	am1.4.1	ea1	ea1	am1.1	am2.1|am1.2|ea1|ea1|am1.4.1|ea1|ea1|am1.1	B3.2
3	am2.1	am1.2	ea1	ea1	am1.4.1	ea1	ea1	—	am2.1|am1.2|ea1|ea1|am1.4.1|ea1|ea1| —	B3.2^a^
4	am2.1	am1.2	ea1	ea1	am1.4.1	ea1	ea1	am1.1	am2.1|am1.2|ea1|ea1|am1.4.1|ea1|ea1|am1.1	B3.2

Sample identification: Sample 1, *Fregata magnificens* (EPI_ISL_17973443); Sample 2, *Fregata magnificens* (EPI_ISL_17973458); Sample 3, *Sula nebouxii* (EPI_ISL_18137626); and Sample 4, *Sula nebouxii* (EPI_ISL_18137671).

a Genotype assignment for Sample 3 was based on seven genome segments because the NS segment was unavailable; consequently, the complete eight-segment genotype was not determined.

### 3.3 Exploratory phylogenetic reconstruction

The exploratory phylogenetic reconstruction included 2,314 H5N1 avian influenza virus sequences belonging to clade 2.3.4.4b and genotype B3.2. Based on genetic proximity, the sequences were distributed among seven major clusters: (1) ANT–CHL–O.S.A.; (2) ARG–BRA–CHL–URY; (3) ARG–BOL–BRA–CHL–PER–URY; (4) ANT–ARG–BRA–CHL–O.S.C.–PER–URY; (5) CHL–ECU–PER; (6) COL–CAN–ECU–O.C.A.–USA; and (7) ANT–CAN–USA (Figure 3).

**Figure 3:**
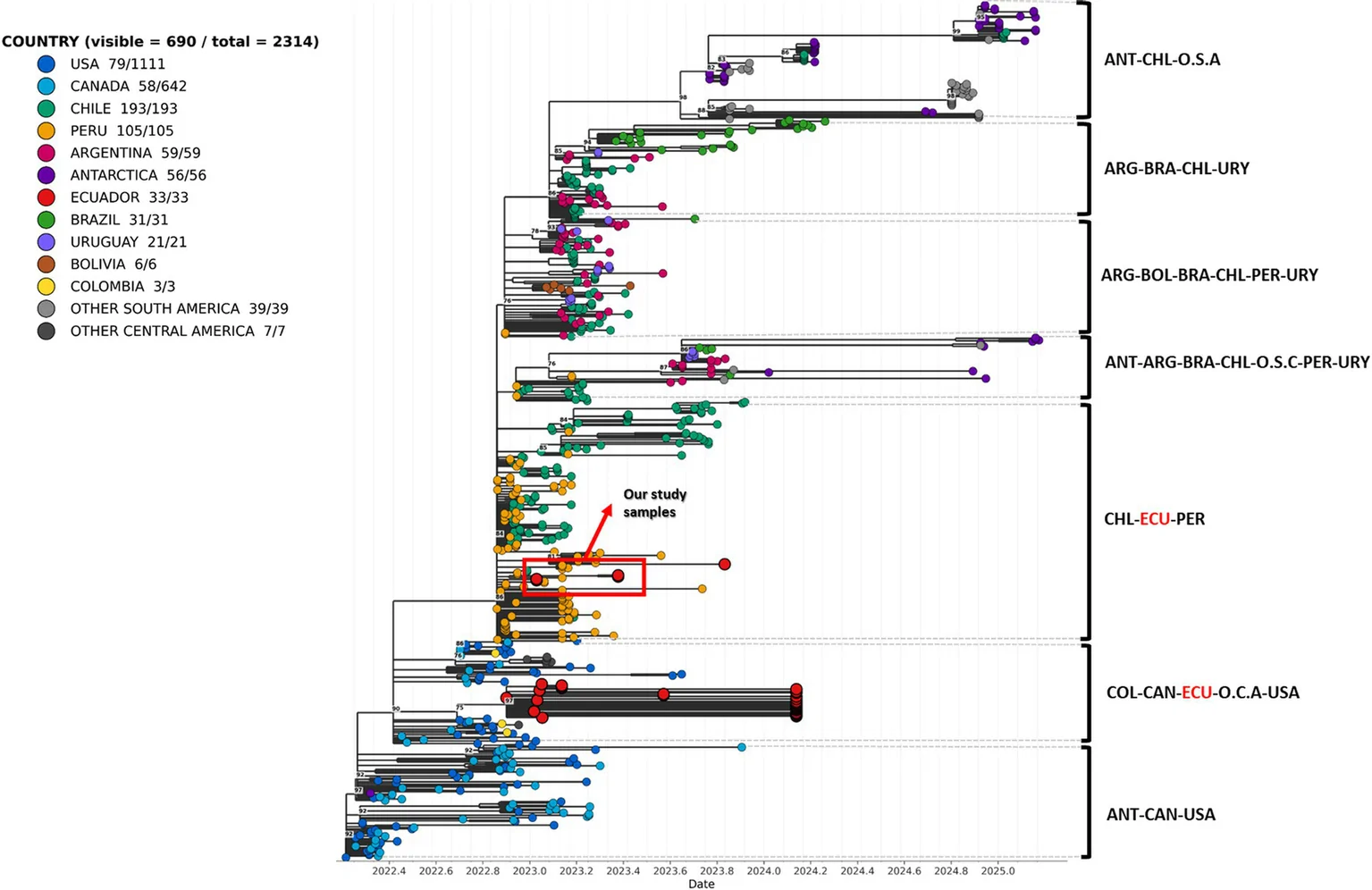
Time-scaled maximum-likelihood phylogeny of highly pathogenic avian influenza A(H5N1) viruses belonging to clade 2.3.4.4b and genotype B3.2. The tree was reconstructed from hemagglutinin sequences and includes 2,314 sequences retained after applying the quality and metadata filters. For visualization purposes, only 690 sequences are displayed in the tree; the remaining sequences were omitted from the figure to improve readability. Ecuador is represented in the clade labels CHL–ECU–PER and COL–CAN–ECU–O.C.A.–USA, indicating phylogenetic groupings containing sequences from Ecuador and the corresponding countries or geographic regions. The red-outlined cluster and arrow identify the sequences generated in the present study. Bootstrap values are shown at selected internal nodes. ANT, Antarctica; ARG, Argentina; BOL, Bolivia; BRA, Brazil; CAN, Canada; CHL, Chile; COL, Colombia; ECU, Ecuador; O.C.A., other Central America; O.S.C., other South America; PER, Peru; URY, Uruguay; USA, United States of America.

Ecuadorian sequences were identified in two distinct clusters. The wild seabird sequences analyzed in this study were grouped within cluster 5 (CHL–ECU–PER), where they were more closely related to sequences from Peru. In contrast, Ecuadorian sequences obtained from domestic birds were located in cluster 6 (COL–CAN–ECU–O.C.A.–USA) and were most closely grouped with sequences from the United States.

For visualization purposes, 690 of the 2,314 sequences were displayed in the phylogenetic tree. The remaining sequences were included in the analysis but were not displayed in the figure.

### 3.4 Whole genome phylogeographic analysis

The time-scaled phylogeny reconstructed from H5N1 sequences available from South America included viruses from Ecuador, Peru, Chile, Argentina, and Uruguay, together with selected sequences from the United States used to provide broader phylogenetic context (Figure 4A). The Ecuadorian sequences were distributed in two distinct, non-monophyletic clusters within the South American phylogeny.

**Figure 4:**
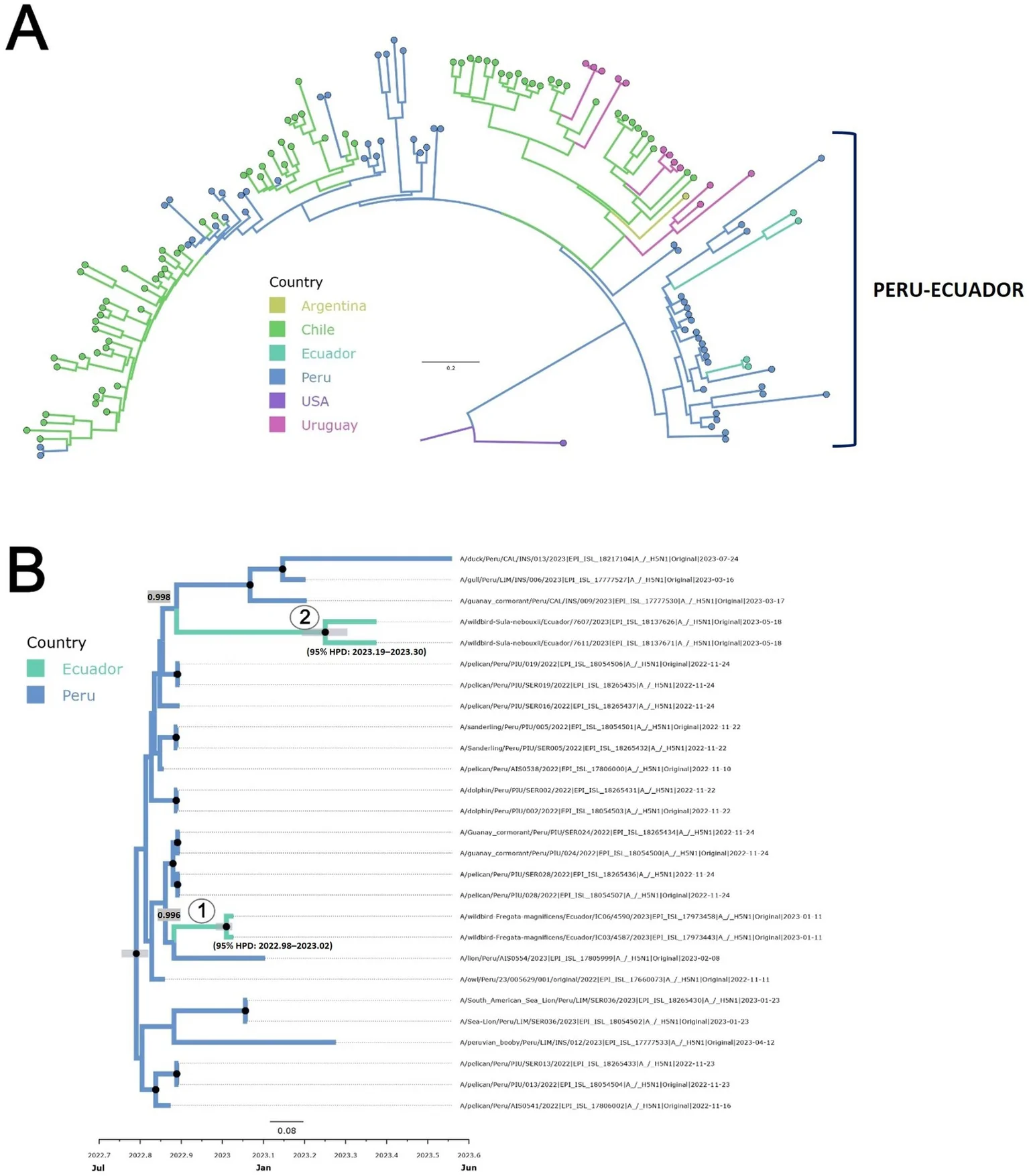
Bayesian phylogenetic reconstruction based on concatenated genome segments of HPAI A(H5N1) clade 2.3.4.4b genotype B3.2 viruses. **(A)** Circular phylogeny showing the geographic distribution of South American sequences. Branches and tips are colored according to the country of detection, and the Peru–Ecuador genetic group is indicated by the bracket. **(B)** Time-scaled phylogenetic reconstruction of the Peru–Ecuador group, highlighting the two Ecuadorian wild-seabird clusters (Clusters 1 and 2). Values shown at the ancestral nodes indicate the posterior probabilities supporting Peru as the most probable ancestral geographic state. Black circles indicate supported nodes, and horizontal gray bars represent the 95% highest posterior density (HPD) intervals of the estimated times to the most recent common ancestors (tMRCAs). Ecuadorian sequences are shown in green and Peruvian sequences in blue.

Both Ecuadorian clusters were nested within lineages predominantly composed of Peruvian sequences (Figure 4B). Discrete phylogeographic reconstruction identified Peru as the most probable ancestral location for the nodes subtending the two Ecuadorian clusters, with an Ancestral State Reconstruction (ASR) cluster 1—0.988 and cluster 2—0.996. The phylogenetic separation of these clusters, together with the independent reconstruction of their ancestral locations, identified two distinct Ecuadorian phylogenetic lineages within the South American dataset.

Cluster 1 comprised two sequences obtained from *F. magnificens*, EPI_ISL_17973443 and EPI_ISL_17973458, both collected in January 2023. The most recent common ancestor (MRCA) was estimated to be 2022 (95% HPD: 2022.98–2023.02) (Figure 4B).

Cluster 2 comprised two sequences obtained from *S. nebouxii*, EPI_ISL_18137626 and EPI_ISL_18137671, both collected in May 2023. These viruses formed a phylogenetically distinct lineage from the *F. magnificens* cluster. The MRCA was estimated to be 2023 (95% HPD: 2023.19–2023.30) (Figure 4B).

### 3.5 Mutations

According to the FluServer analysis, 29 amino acid substitutions with previously reported biological significance were identified across five of the eight genomic segments analyzed (Table 3). No relevant substitutions were detected in PB2, PA, or NP. The substitutions identified by FluServer are described below by protein. These annotations represent associations reported for equivalent positions in previous studies and should not be interpreted as phenotypic effects experimentally demonstrated in the Ecuadorian viruses.

**Table 3:** Amino acid substitutions identified in the four Ecuadorian A(H5N1) genomes and their reported biological significance.

Gene	aa change	Known equivalent position	Biological significance level	Effect	Reference residue	Sample 1	Sample 2	Sample 3	Sample 4
PB2	—	—	—	—	—	—	—	—	—
PB1	P598L	P598L from series V473L	1	Polymerase activity of the H5N1 virus, especially in mammalian cells	P	L	L	L	X
PA	—	—	—	—	—	—	—	—	—
HA	D110S	D94N	1	Host specificity shift. Able to enhance HA-mediated membrane fusion in mammalian cells	D	S	—	S	S
	S139P	S123P from series E75K	1	Produced variable increases in SAα2,6Gal recognition	S	P	—	P	—
	D142E	D126N	1	Virulence, antigenic drift/escape mutant	D	N	—	N	—
	A143T	A131D	1	Antigenic drift/escape mutant	A	D	—	D	—
	S145L	S145N/L129V	1	Antigenic drift/escape mutant, host specificity shift	S	L	—	L	—
	S149A	S137A	3	Host specificity shift. Increased α2,6-SA binding and decreased α2,3-SA binding	S	A	—	A	—
	H154Q	N142K from series N142K, K156N	1	Antigenic drift/escape mutant	H	Q	—	Q	—
	S157P	S145F	1	Virulence, antigenic drift/escape mutant	S	P	—	P	—
	S171D/S171N	S159N	1/3	Host specificity shift. Increased binding to α2,6-linked sialic acids without loss of α2,3-linked binding	S	D	—	N	—
	D199N	D187G	1	Host specificity shift	D	N	—	N	—
	Q208K	Q192R	1	Host specificity shift	Q	K	—	K	—
	V226A	V210I	1	Host specificity shift	V	A	—	A	A
	K234Q	K222E	1	Virulence. Increased organ tropism	K	Q	—	Q	Q
	S239R	R205I	1	Virulence, antigenic drift/escape mutant	S	R	—	R	R
	Q338L	S339P from series R233K	1	Mild drug resistance	Q	L	L	L	L
NP	—	—	—	—	—	—	—	—	—
NA	G354S	D354G from series H275Y	1	Strong drug resistance	G	S	S	—	—
	S364G	S367N	1	Related to antigenic drift/escape mutant	S	—	—	G	G
M1/M2	K101R	R101S from series R101S	1	Virulence	K	R	R	R	R
	S31N	S31N	3	Strong drug resistance	S	—	—	—	N
NS1	A42S	P42S	1	Virulence	A	S	S	—	S
	Y103F	F103L	1	Virulence	Y	F	F	—	F
	I205S	N200S	1	Virulence	I	S	S	—	S
	Y221K	C-terminus (RSEV)	1	Virulence	Y	K	K	—	—
	A223E	C-terminus (RSEV)	1	Virulence	A	E	E	—	—
	K224R	C-terminus (RSEV)	1	Virulence	K	R	R	—	—
	R225T	C-terminus (RSEV)	1	Virulence	R	T	T	—	—
	V226I	C-terminus (RSEV)	1	Virulence	V	I	I	—	—
NS2	S48A	T47A	1	Virulence	S	A	A	—	A

Biological significance level: 1, mutation at a site known to be involved in drug binding or host-cell specificity; 2, mutation that creates or eliminates a potential glycosylation site; 3, mutation that alters viral virulence, causes strong drug resistance, or reverses the effect of a premature stop codon in PB1-F2.

Samples: Sample 1, *Fregata magnificens* (EPI_ISL_17973443); Sample 2, *Fregata magnificens* (EPI_ISL_17973458); Sample 3, *Sula nebouxii* (EPI_ISL_18137626); Sample 4, *Sula nebouxii* (EPI_ISL_18137671).

In HA, 15 substitutions were identified: D110S, S139P, D142E, A143T, S145L, S149A, H154Q, S157P, S171D/S171N, D199N, Q208K, V226A, K234Q, S239R, and Q338L. Four substitutions (D142E, S157P, K234Q, and S239R) were annotated as being associated with increased virulence, while six (D142E, A143T, S145L, H154Q, S157P, and S239R) were associated with antigenic drift or immune escape. Eight substitutions (D110S, S139P, S145L, S149A, S171D/S171N, D199N, Q208K, and V226A) were associated with changes in host specificity or receptor-binding preference. HA-D110S was additionally annotated as being associated with enhanced HA-mediated membrane fusion in mammalian cells. HA-Q338L, annotated as being associated with mild antiviral resistance, was present in all four samples. Most of the remaining HA substitutions were detected in samples 1 and 3, while V226A, K234Q, and S239R were also detected in sample 4.

In NA, two substitutions were identified. NA-S364G, detected in samples 3 and 4, was annotated as being associated with antigenic drift or immune escape. NA-G354S, detected in samples 1 and 2, was annotated as being associated with strong antiviral resistance. In PB1, P598L was detected in samples 1–3 and was annotated as being associated with increased polymerase activity in mammalian cells. In the M segment, two substitutions were identified. MP1-K101R, detected in all four samples, was annotated as being associated with increased virulence. MP2-S31N, detected only in sample 4, was annotated as being associated with strong antiviral resistance.

Finally, nine substitutions annotated as being associated with increased virulence were identified in NS1/NS2. The NS1 substitutions A42S, Y103F, and I205S were detected in samples 1, 2, and 4. NS1-Y221K, A223E, K224R, R225T, and V226I were detected in samples 1 and 2, whereas NS2-S48A was detected in samples 1, 2, and 4.

## 4 Discussion

This study presents the genomic characterization of highly pathogenic avian influenza A(H5N1) clade 2.3.4.4b viruses detected in frigatebirds (*F. magnificens*) and blue-footed boobies (*S. nebouxii*) from coastal Ecuador during the early phase of the South American epizootic. These cases represent confirmed detections of avian influenza in wild birds in Ecuador. Although all four viruses shared the B3.2 genome constellation, they were distributed into two temporally and phylogenetically distinct clusters, both closely related to viruses detected in Peru. This pattern indicates genetic heterogeneity among the Ecuadorian wild seabird viruses and is compatible with independent introduction events; however, the available data do not allow the number or direction of introduction events to be determined conclusively. The high sequencing depth and extensive recovery of the viral segments provided sufficient resolution for HA clade assignment, whole-genome genotyping, and phylogeographic inference.

All genomes reported in this study were assigned to H5 clade 2.3.4.4b. This clade has undergone rapid intercontinental expansion. Following its transatlantic introduction from Europe, the virus was detected in North America in late 2021, became established in wild birds, reassorted with locally circulating avian influenza viruses, and subsequently spread into Central and South America during 2022 (Caliendo et al., 2022; Alkie et al., 2023; Youk et al., 2023b; Leguia et al., 2023). The detection of clade 2.3.4.4b viruses in Ecuador adds to the evidence of H5N1 dissemination along the eastern Pacific coast of South America and highlights the importance of continued genomic surveillance in coastal and migratory birds.

Our four genomes were assigned to genotype B3.2, with the segment constellation am2.1|am1.2|ea1|ea1|am1.4.1|ea1|ea1|am1.1. This genotype arose through reassortment between the Eurasian-origin H5N1 virus introduced into North America and low-pathogenicity avian influenza viruses circulating among North American wild birds, resulting in a mosaic genome containing Eurasian and North American lineage segments (Youk et al., 2023a; Uhart et al., 2024). B3.2 was subsequently detected during the early southward expansion of H5N1 along the Pacific coast of South America. In Peru, viruses with this reassortant genome constellation were identified in pelicans, cormorants, sanderlings, South American sea lions, and a dolphin during mortality events beginning in November 2022 (Leguia et al., 2023). Peruvian viruses also formed a monophyletic group with sequences from Chile, consistent with regional dissemination along the eastern Pacific coast (Castro-Sanguinetti et al., 2024).

The exploratory phylogenetic reconstruction shown in Figure 3 provided the initial broader genetic context for the Ecuadorian detections. Among the 2,314 H5N1 clade 2.3.4.4b genotype B3.2 sequences analyzed, seven genetically defined clusters were identified. Despite sharing the same genotype assignment, the Ecuadorian sequences were distributed across two distinct genetic contexts. The wild seabird viruses analyzed in this study clustered within the CHL–ECU–PER group and were most closely related to viruses from Peru. In contrast, sequences previously obtained from domestic birds in Ecuador were located in the separate COL–CAN–ECU–O.C.A.–USA group and showed a closer genetic association with viruses from the United States. This pattern suggests that the B3.2 viruses detected in Ecuador belong to distinct phylogenetic backgrounds. The separation between the wild seabird and domestic-bird sequences is compatible with independent introduction events through different geographic or ecological pathways, although alternative explanations, including unsampled transmission links and regional sampling imbalance, cannot be excluded (Lemey et al., 2020).

The subsequent focused phylogenetic reconstruction further resolved the relationships among the four Ecuadorian wild seabird viruses. The viruses did not form a single monophyletic group: Cluster 1, as designated in Figure 3B, comprised the two *F. magnificens* viruses and had a most recent common ancestor estimated in early January 2023, whereas Cluster 2 comprised the two *S. nebouxii* viruses and had a most recent common ancestor estimated in early April 2023. These estimates represent the inferred timing of the common ancestry of each sampled cluster and should not be interpreted as exact dates of viral introduction into Ecuador. Peru was reconstructed as the most probable ancestral geographic state for both clusters, with posterior probabilities exceeding 0.98. However, this inference may have been influenced by uneven regional sampling and does not exclude unsampled intermediate transmission events or additional introduction pathways. Given that only four Ecuadorian genomes were analyzed, the two distinct Ecuadorian lineages represent the phylogenetic pattern supported by the available genomic data and should not be interpreted as a definitive estimate of the total number of introduction events.

The ecology of the affected hosts provides a plausible framework for repeated exposure to viruses circulating along the eastern Pacific coast. Both *F. magnificens* and *S. nebouxii* are colonial seabirds that use coastal or island resting and breeding sites and may overlap with multispecies assemblages in productive marine feeding areas. Frigatebirds can forage across extensive marine areas (Giambalvo et al., 2022), whereas breeding blue-footed boobies in northern Peru have been documented travelling tens of kilometers from their colonies during individual foraging trips (Zavalaga et al., 2008). Colonial aggregation, overlap at feeding and resting sites, and contact with infected birds or contaminated environments may facilitate influenza transmission among susceptible seabirds (Lang et al., 2016; Klaassen and Wille, 2023; Couty et al., 2026). However, the present data do not identify which species introduced, amplified, or maintained the virus. Instead, they indicate that both hosts were affected within broader coastal circulation and that their viruses were associated with distinct phylogenetic histories, with no evidence of host-associated reassortment in this limited dataset.

FluServer identified 29 amino acid substitutions previously associated with virulence, receptor specificity, antigenic change, polymerase activity, or antiviral resistance. However, these findings represent literature-based computational annotations rather than experimentally validated phenotypes in the Ecuadorian B3.2 viruses, because effects reported in other subtypes, viral backbones, hosts, or experimental systems may not be directly transferable (Davis et al., 2014; Suttie et al., 2019). In the PB2 segment, neither E627K nor D701N was detected, distinguishing the Ecuadorian viruses from some H5N1 viruses identified in South American marine mammals that carried markers previously associated with mammalian adaptation (Uhart et al., 2024; Tomás et al., 2024; Pardo-Roa et al., 2025). Similarly, the biological relevance of HA-S171D should be interpreted cautiously, because receptor recognition and transmissibility depend on combinations of receptor-binding-site residues, receptor avidity, HA stability, and HA–NA functional balance rather than on a single substitution (Yamada et al., 2006; Imai et al., 2012; Herfst et al., 2012). Moreover, the absence of HA-Q226L and HA-G228S, using H3-equivalent numbering, provides no molecular evidence of the canonical shift toward preferential recognition of human-type α2,6-linked sialic acids. In the M segment, M2-S31N was detected; unlike most of the other annotations, this substitution is strongly supported as a determinant of resistance to amantadine and rimantadine (Hurt et al., 2007; Pielak et al., 2009; Govorkova et al., 2013). Finally, the NS1 and NS2 annotations should not be interpreted collectively as an additive virulence score, because their effects remain dependent on the viral background, host species, and experimental context (Hale et al., 2008; Ayllon and García-Sastre, 2015). Overall, these molecular findings identify candidates for future functional investigation but do not, by themselves, demonstrate enhanced mammalian adaptation, pathogenicity, or human-to-human transmissibility in the viruses analyzed here. Nevertheless, sustained circulation in wildlife increases the opportunity for adaptive mutations and reassortment events that could alter viral fitness, host range, transmissibility, or virulence. Continued genomic surveillance is therefore essential for the early detection of emerging variants with potential relevance to animal and human health.

The main concern is therefore not any single molecular marker, but the possibility of repeated H5N1 introductions into connected wildlife populations, which could increase opportunities for host switching, mutation, reassortment, and selection. In coastal Ecuador, contact with infected animals, carcasses, fecal material, or contaminated environments may also create exposure risks at wildlife–domestic animal–human interfaces. Joint FAO–WHO–WOAH assessments identify direct or indirect contact with infected animals or contaminated environments as the main context for reported human A(H5N1) infections and classify occupational risk as low to moderate, depending on exposure and mitigation measures (FAO, WHO and WOAH, 2024). The presence of susceptible and conservation-priority bird populations in the Galápagos further supports coordinated surveillance. However, substantial genetic differentiation and long-term isolation of Galápagos frigatebirds from continental populations have been reported, and the present study did not directly assess viral connectivity between continental Ecuador and the archipelago (Hailer et al., 2011). Surveillance should therefore combine investigations of unusual morbidity and mortality with targeted active sampling of wild birds, poultry, and marine mammals, as mortality-based surveillance alone may underestimate viral circulation.

This study has some limitations. Only four genomes from two seabird species were analyzed; therefore, the two identified phylogenetic lineages should not be interpreted as a definitive or minimum estimate of the number of H5N1 introduction events into Ecuador. Phylogeographic inference depended on the temporal, geographic, and host distribution of publicly available sequences and may have been affected by regional sampling imbalance (Lemey et al., 2020). The incomplete recovery of the NS segment in one sample prevented complete assessment of NS1/NS2 associated substitutions. In addition, functional inferences were based on computational annotation and previously described molecular markers, as no phenotypic experiments were performed to validate the biological significance of the identified mutations.

Despite these limitations, the genomic data revealed two distinct phylogenetic lineages of H5N1 clade 2.3.4.4b genotype B3.2 among Ecuadorian coastal wild seabirds during early 2023. Their close phylogenetic relationship with Peruvian viruses places these detections within a broader pattern of regional circulation along the eastern Pacific coast, while the separation of the two Ecuadorian clusters shows that a shared genome constellation should not be interpreted as evidence of a single transmission chain. Although the observed phylogenetic structure is compatible with independent introduction events, the limited number of Ecuadorian genomes and uneven regional sampling prevent definitive conclusions regarding the total number or direction of introductions. Coordination among wildlife, veterinary, environmental, and public-health authorities within a One Health framework will therefore be essential for protecting biodiversity, animal health, and occupationally exposed populations.

## Data Availability

The datasets presented in this study can be found in online repositories. The names of the repository/repositories and accession number(s) can be found in the article/Supplementary material.

## Footnotes

1
http://flusurver.bii.a-star.edu.sg
