# Supplementary material for: Genomic characterization of highly pathogenic avian influenza A(H5N1) clade 2.3.4.4b into wild seabirds from coastal Ecuador
Source: Front Microbiol. 2026 Sep 25;17:1953002. doi: 10.3389/fmicb.2026.1953002 (PMC13649745; doi:10.3389/fmicb.2026.1953002)
Supplement: Supplementary file 1 [file Table_1.DOCX]

Supplementary Material

# Supplementary Table

**Supplementary Table 1.** Metadata for the four A(H5N1) viruses detected in wild seabirds from coastal Ecuador in 2023**.**

| **GISAID accession** | **Host species** | **Domestic status** | **Specimen source** | **Health status** | **Collection location** | **Collection date** | **Coordinates (lat, long)** |
| --- | --- | --- | --- | --- | --- | --- | --- |
| EPI_ISL_17973443 | *Fregata magnificens* | Wild | Tracheal swab | Dead | South America / Ecuador / Manabí / San Vicente | 2023-01-11 | -0.58998, -80.40704 |
| EPI_ISL_17973458 | *Fregata magnificens* | Wild | Tracheal swab | Sick | South America / Ecuador / Manabí / San Vicente | 2023-01-11 | -0.58998, -80.40704 |
| EPI_ISL_18137626 | *Sula nebouxii* | Wild | Tracheal swab | Sick | South America / Ecuador / Guayas / General Villamil Playas | 2023-05-18 | -2.6388, -80.3892 |
| EPI_ISL_18137671 | *Sula nebouxii* | Wild | Tracheal swab | Sick | South America / Ecuador / Guayas / General Villamil Playas | 2023-05-18 | -2.6388, -80.3892 |
